# Crosstalk between 6-methyladenine and 4-methylcytosine in *Geobacter sulfurreducens* exposed to extremely low-frequency electromagnetic field

**DOI:** 10.1016/j.isci.2024.110607

**Published:** 2024-07-27

**Authors:** Zhenhua Shi, Yingrong Zhang, Wanqiu Chen, Zhen Yu

**Affiliations:** 1 College of Resources and Environment, Fujian Agriculture and Forestry University, 15 Shang Xia Dian Road, Cang Shan District, Fuzhou, Fujian 350002, China; 2 Fujian Provincial Key Laboratory of Medical Analysis, Fujian Academy of Medical Sciences, 7 Wu Si Road, Gu Lou District, Fuzhou, Fujian 350001, China

**Keywords:** Exposure, Electromagnetic field, Epigenetics, Microbial genomics, Molecular microbiology

## Abstract

4-Methylcytosine (4mC) and 6-methyladenine (6mA) are the most prevalent types of DNA modifications in prokaryotes. However, whether there is crosstalk between 4mC and 6mA remain unknown. Here, methylomes and transcriptomes of *Geobacter sulfurreducens* exposed to different intensities of extremely low frequency electromagnetic fields (ELF-EMF) were investigated. Results showed that the second adenine of all the 5′-GTACAG-3′ motif was modified to 6mA (M-6mA). For the other 6mA (O-6mA), the variation in their distance from the neighboring M-6mA increased with the intensity of ELF-EMF. Moreover, cytosine adjacent to O-6mA has a much higher probability of being modified to 4mC than cytosine adjacent to M-6mA, and the closer an unmodified cytosine is to 4mC, the higher the probability that the cytosine will be modified to 4mC. Furthermore, there was no significant correlation between DNA methylation and gene expression regulation. These results suggest a reference signal that goes from M-6mA to O-6mA to 4mC.

## Highlights

• 4mC modification pattern follows the law of a power function
• DNA methylation modification pattern is established at the genome-wide level
• 4mC and 6mA have crosstalk during DNA methylation
• Both level and pattern of DNA methylation not serve for gene expression regulation

## Graphical abstract

**Figure:**
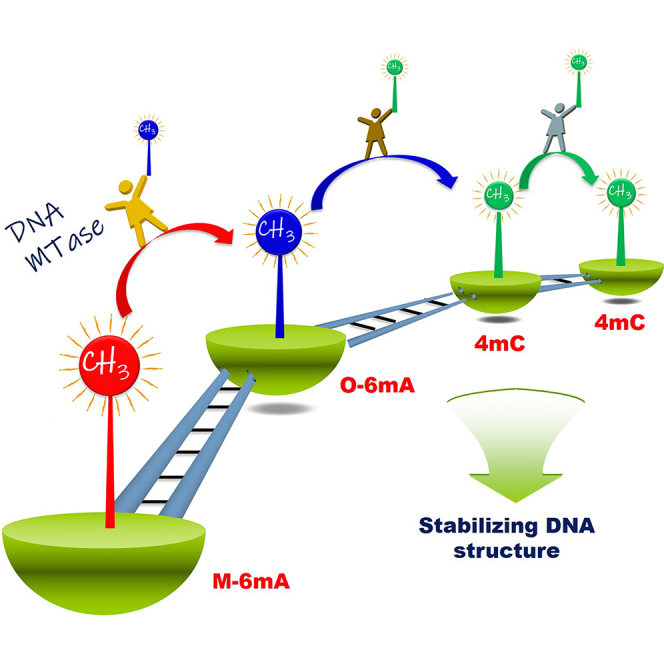

## Introduction

In prokaryotes, DNA methylation is widespread and has profound biological consequences.^1^^,^^2^^,^^3^ Unlike in plant, animal, or fungal models where DNA methylation occurs only at the fifth position of cytosine, prokaryotes exhibit two prevalent types of DNA modifications: 4-methylcytosine (4mC) and 6-methyladenine (6mA).^4^^,^^5^^,^^6^^,^^7^^,^^8^ Both 4mC and 6mA have been reported to be associated with DNA replication and repair, and gene expression regulation.^9^^,^^10^^,^^11^^,^^12^^,^^13^ The overlapping effects of 4mC and 6mA suggest that there may exist crosstalk between these two types of methylation, which need to be explored.

For most eukaryotes, oxygen is the only terminal electron acceptor in the respiratory electron transport chain. On the contrary, *Geobacter* species could utilize a wide range of electron acceptors, including Fe(III), Co(III), U(VI), Tc(VII), fumarate, and humic acids.^14^^,^^15^^,^^16^^,^^17^^,^^18^^,^^19^ Our previous study has shown that 4mC and 6mA modifications are widespread in the whole genome of *Geobacter sulfurreducens*, and different electron acceptor cultures have different 4mC and 6mA methylation patterns.^20^ Thus, in this study, *G. sulfurreducens* was selected as a model organism to explore the crosstalk of 6mA and 4mC.

Accumulating studies have demonstrated that the DNA methylation patterns of a specific gene or the whole genome could be changed under the influence of environment stimuli.^21^^,^^22^^,^^23^^,^^24^ Extremely low-frequency electromagnetic fields (ELF-EMF) are ever-present due to the extensive use of and increasing worldwide demand for electricity, and it has been established that small and very small doses of ELF-EMF are affecting living cells and organisms, including bacteria. For example, exposure to ELF-EMF produced a significant change of morphotype,^25^ growth rate,^26^ cell adhesion,^27^ membrane potential,^28^ and conformation of chromatin.^29^ Besides, ELF-EMF is an environmental stimulus having the following characteristics: non-ionizing and unable to induce thermal effects, easy control and no metabolic byproduct, and has a dose-dependent influence on the production of metabolic electrons flowing along the electron transport chain.^30^ Thus, in the present study, genome-wide 6mA and 4mC of *G. sulfurreducens* exposed to different intensities of ELF-EMF were examined by third-generation, single-molecule real-time (SMRT) DNA sequencing. Moreover, in order to explore the roles of 4mC and 6mA in gene expression regulation, transcriptome profiles were generated by RNA sequencing (RNA-seq) on the same materials.

## Results and discussion

### Methylome analysis of *G. sulfurreducens* exposed to different ELF-EMF intensities

**Figure 1:**
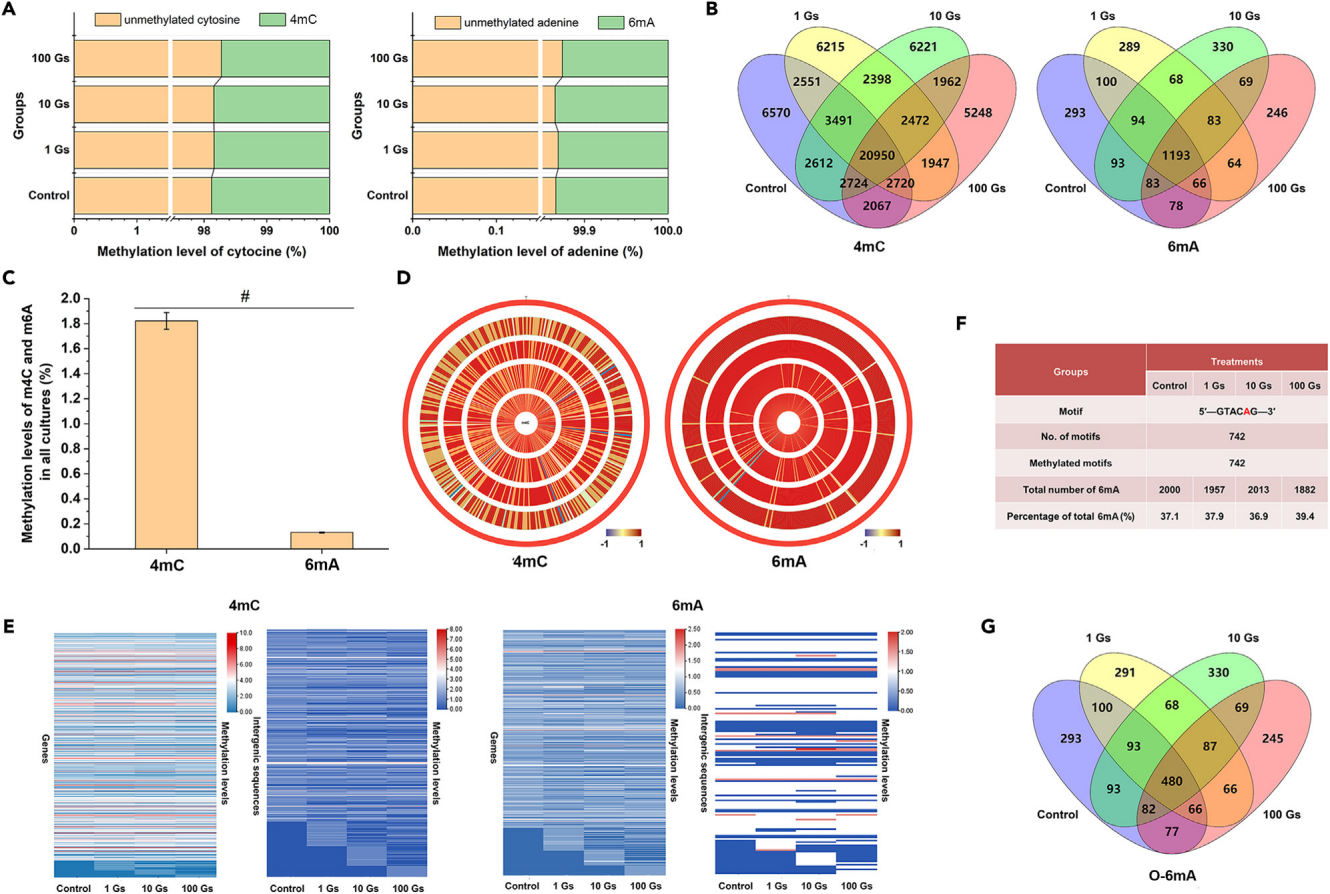
Methylome analysis of *G sulfurreducens* exposed to different ELF-EMF intensities (A) Percentages of 4mC and 6mA in the genome to total cytosine and adenine. (B) Overlap analysis of 4mC and 6mA. (C) Methylation levels of 4mC and 6mA in all samples (*p* < 0.001). (D) Circular display of differentially methylated region (DMR) distribution across the genome. The outermost circle represents the genome. The red and blue regions denote hypermethylated and hypomethylated DMRs, respectively. (E) Heat maps showing 4mC and 6mA methylation levels in gene and intergenic sequences. (F) Summary of detected 6mA-methylated motifs across the genome. (G) Overlap analysis of O-6mA.

Methylome analysis showed that among the total genomic cytosines, 1.88% (43,685) was methylated in the control samples, whereas 1.84% (42,744), 1.84% (42,830), and 1.72% (40,090) were methylated in the samples treated with 1, 10, and 100 G ELF-EMF, respectively. Among the total genomic adenosines, 0.13% (2,000) were methylated in the control samples, whereas 0.13% (1,957), 0.14% (2,013), and 0.13% (1,882) were methylated in the samples treated with 1, 10, and 100 G ELF-EMF, respectively (Figure 1A). These results suggest that there are no distinct influences of ELF-EMF on genomic 4mC or 6mA methylation level. Interestingly, overlap analysis of the location of these modifications in the genome showed that only ∼30% of 4mC had the same gene locus in all samples, while the value was ∼38% for 6mA (Figure 1B). This result suggests that the distribution patterns of 4mC or 6mA methylation varied among these samples. In other words, many methylated sites in one sample are no longer methylated in other samples, and sites that are not methylated in one sample may be methylated in other samples. It has been reported that DNA methylation responds to environmental variations by changing the conformation of DNA, which plays an important role in protein-DNA interactions.^31^^,^^32^ Thus, variations of 4mC or 6mA methylation pattern may be involved in the response of *G. sulfurreducens* to ELF-EMF.

Besides, the number of 4mC was 20 times more than that of 6mA in all samples (Figure 1C). Circular visualization and heatmaps showed that 4mC and 6mA are distributed differently in different samples, both in gene sequences and intergenic sequences (Figures 1D and 1E). Motif analysis revealed one DNA methyltransferase specificity, 5′-GTACAG-3′, and the second adenine of this motif was modified to 6mA in all samples (Figure 1F). The 6mA located in this motif is termed M-6mA, and their amount is ∼37% of the total 6mA in all samples. For the other 6mA, termed O-6mA, ∼20% have the same location in the genome of all the samples (Figure 1G). These results revealed that *G. sulfurreducens* have different 4mC and O-6mA methylation patterns under different ELF-EMF exposure conditions.

### Methylation patterns of 4mC/6mA and their crosstalk

Considering the large number of differentially methylated sites (more than 30%) in different samples and the important role of DNA methylation in bacterial environmental adaption, we wondering if there is a common rule followed by DNA methylation. It is known that DNA is a molecule composed of two polynucleotide chains that coil around each other to form a double helix and a strand usually circles the axis of the double helix once every 10.4 base pairs. The structural characteristics of DNA suggest that DNA methyltransferase may have modification preferences for cytosine or adenine with spatial differences in double helix structure. To test this hypothesis, the distances (unit: bp) between all adjacent 4mC/6mA sites (4mC and 4mC, 4mC and 6mA, 6mA and 6mA) throughout the genome were calculated, and the frequency of each calculated distance value was counted and compared.

**Figure 2:**
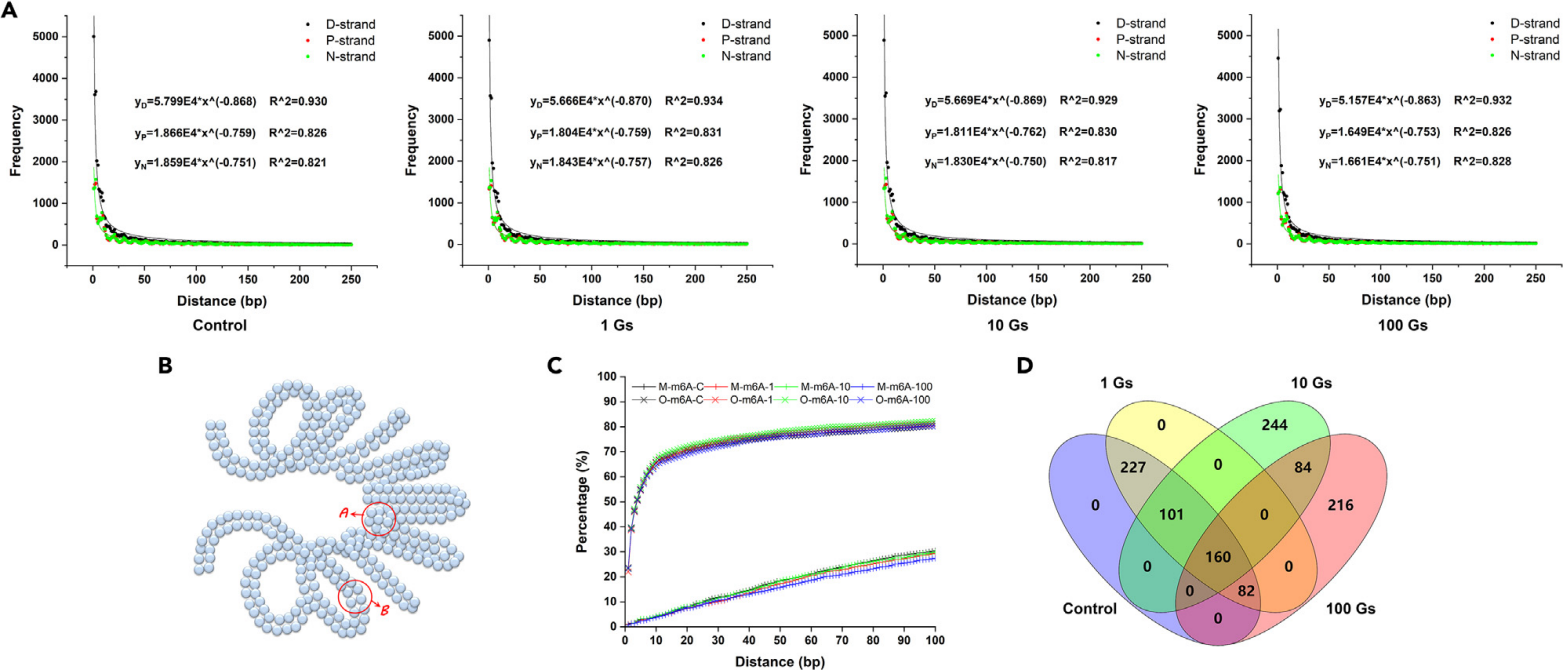
Methylation patterns of 4mC and 6mA and their relationship (A) Distances less than and equal to 250 bp were divided into 25 equal bins, and the fitting formulae of frequency-bin curves were given in corresponding figures. P-, N-, and D-strands indicate positive, negative, and double strands, respectively. (B) Schematic diagram for the effect of DNA superhelices structure on the space for DNA methylation. (C) Cumulative of the percentage of 4mC within the distances from 4mC to O-6mA and from 4mC to M-6mA. (D) Overlap analysis of O-6mA neighboring M-6mA.

As shown in Figure 2A, the distances (x axis) of adjacent two 4mC modification and their frequencies (y axis) followed a power function, *y* = *ax*^*b*^ (*a*>0, *b* < 0), in all samples. Specifically, the smaller the distance value, the higher the frequency. In other words, 4mC modification is a biochemical reaction with a distance effect following a pattern that as the distance of a specific cytosine to the nearest 4mC declines, the probability of this specific cytosine methylation goes up. This phenomenon occurs not only in calculations based on double strands of DNA, but also in calculations based on any single strand. Similar result was also shown in *G. sulfurreducens* grown with different types of electron acceptors (hydrous ferric oxide, Fe(III) citrate, and fumarate).^33^ It is worth mentioning that if follow this pattern without any restriction, all the genomic cytosine would be methylated eventually. However, more than 98% of cytosines in the genome were not modified in all samples. One of the most likely explanations is the DNA superhelices structure in cells resulting in limit number of sites with enough space for methylation, as shown in Figure 2B, where the bases in region “B” are more likely to be methylated than those in region “A.”

For exploring whether 6mA modification involved in 4mC modification, we calculated the distance (unit: bp) between O-6mA/M-6mA and the adjacent 4mC. The results showed that more than 50% of O-6mA had a distance of less than 5 bp from adjacent 4mC (Figure 2C). Of these O-6mA, more than 20% had a distance of only 1 bp. For M-6mA, the values were less than 5% and 1%, respectively. The results suggest that cytosine closer to O-6mA is more likely to be modified to 4mC. Considering that the number of 4mC is much larger than the number of 6mA and that O-6mA modifications vary with changes in the intensity of the ELF-EMF, our results further suggest that O-6mA is a reference for 4mC modification.

Next, we calculated and compared the distances between all adjacent M-6mA and O-6mA. Overlap analysis showed that, the samples treated with 1 G ELF-EMF had the same amount of each distance compared to the control group, while more than 40% of the 10 G and 100 G ELF-EMF treated samples had different distances. The value was ∼40% for samples treated with 10 G ELF-EMF compared to samples treated with 100 G ELF-EMF (Figure 2D). The results showed that the position of O-6mA relative to M-6mA in the genome varies with changes in the intensity of the ELF-EMF. Considering that the amount of M-6mA is the same in all samples and the location in the genome is fixed, our results further suggest that M-6mA is a reference for O-6mA modification.

These results suggest that 4mC and 6mA have crosstalk during DNA methylation in the process of DNA methylation, that is, DNA methyltransferase uses M-6mA as the reference to modify their neighboring adenine into O-6mA, then uses O-6mA as the reference to modify their neighboring cytosine into 4mC, and further uses 4mC as the reference to modify their neighboring cytosine into 4mC following the law of power function. Given the differences in the amount of M-6mA, O-6mA, and 4mC in each sample, the process of DNA methylation should be a cascade reaction at the genomic level. It has been reported that DNA methylation predominantly occurs at CpG dinucleotides across plant^34^ and mammal genomes,^23^ also suggest that DNA methylation should be considered as a modification at the genomic level instead of genetic level.

### Transcriptome analysis of *G. sulfurreducens* exposed to different ELF-EMF intensities

**Figure 3:**
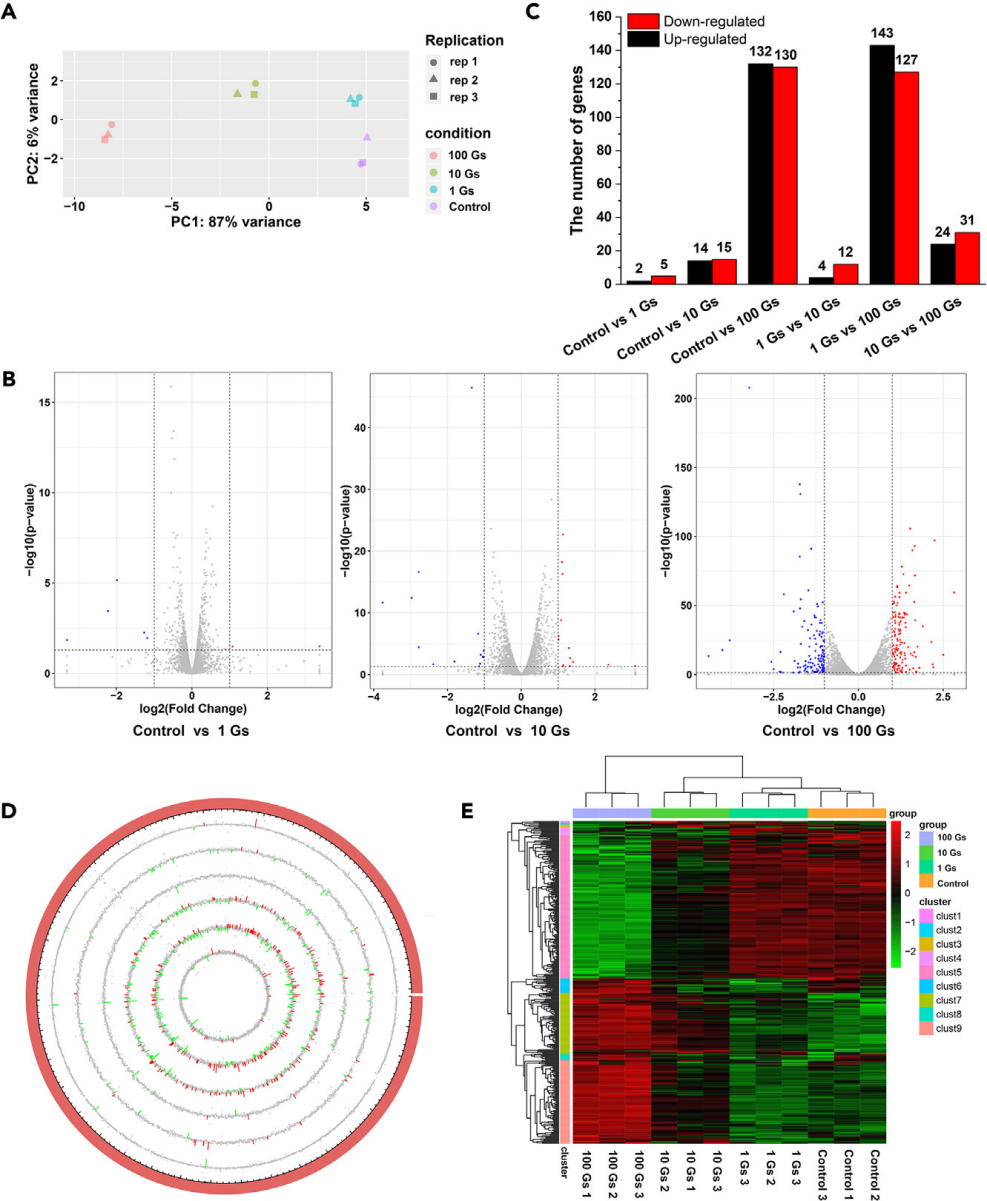
Transcriptome analysis of *G sulfurreducens* exposed to different ELF-EMF intensities (A) PCA analysis. (B) Volcano plot of DEGs. (C) Number of DEGs. (D) Circular display of DEG distribution across the genome of *G. sulfurreducens*. The outermost circle represent the genome of *G. sulfurreducens*. From outside to inside are the follows: control vs. 1 G, control vs. 10 G, control vs. 100 G, 1 G vs. 10 G, 1 G vs. 100 G, and 10 G vs. 100 G. (E) Hierarchical clustering.

For 4mC and 6mA have been reported to be associated with gene expression regulation,^9^^,^^10^^,^^11^^,^^12^^,^^13^ transcriptome profiles were then subsequently examined on the same materials used for methylome analysis. A total of 3,403 coding sequences were annotated in the *G*. *sulfurreducens* genome. Among which, 3,381 genes were represented in the RNA-seq study. Six genes were randomly selected and quantified by qRT-PCR to validate the expression pattern of differentially expressed genes (DEGs) from RNA-seq data. The results indicated the concordant expression patterns of these genes by RNA-seq data and qRT-PCR analyses. The Pearson’s correlation coefficient was 0.888 (*p* = 0.001, *rec*A as the internal reference) or 0.834 (*p* = 0.005, *gap*A as the internal reference), respectively, indicating the high reliability and accuracy of the transcriptomic data analyses (Table S1). Principal-component analysis (PCA) revealed a noticeable separation between the exposure and control groups, and the separation became larger with the increase in ELF-EMF intensity (Figure 3A). Besides, the number of DEGs in the comparison of the exposure and control groups also increased with the increase in ELF-EMF intensity (Figures 3B–3E; Figure S1).

Functional annotation of DEGs showed that, as the intensity of the ELF-EMF increase, DEGs were significantly involved in biological process including “protein secretion by the type II secretion system (GO:0015628),” “protein transport across the cell outer membrane (GO:0098776),” and “cation transport (GO:0006812)”; cellular component including “outer membrane-bounded periplasmic space (GO:0030288),” “periplasmic space (GO:0042597),” and “type II protein secretion system complex (GO:0015627)”; molecular function including “intramolecular oxidoreductase activity (GO:0016861),” “intramolecular oxidoreductase activity (GO:0016860),” and “adenylyltransferase activity (GO:0008882)” (Table S2). Moreover, two pathways of “purine metabolism (ko00230)” and “one carbon pool by folate (ko00670)” were significantly enriched for genes associated with ELF-EMF exposure (Table S3). Membranes are the first line of defense of cells.^35^^,^^36^ For bacteria, the electron transport system are mainly distributed on/in the membrane. Previous studies have determined that the potential of the membrane and the current flowing along the electron transport chain were all changed under ELF-EMF exposure.^28^^,^^30^ Our results were consistent with those studies by showing that DEGs significantly enriched in biological process and cellular component associated with membrane, further corroborated the effect of ELF-EMF on membrane at the transcription level.

### Correlation of gene expression with O-6mA and 4mC

**Figure 4:**
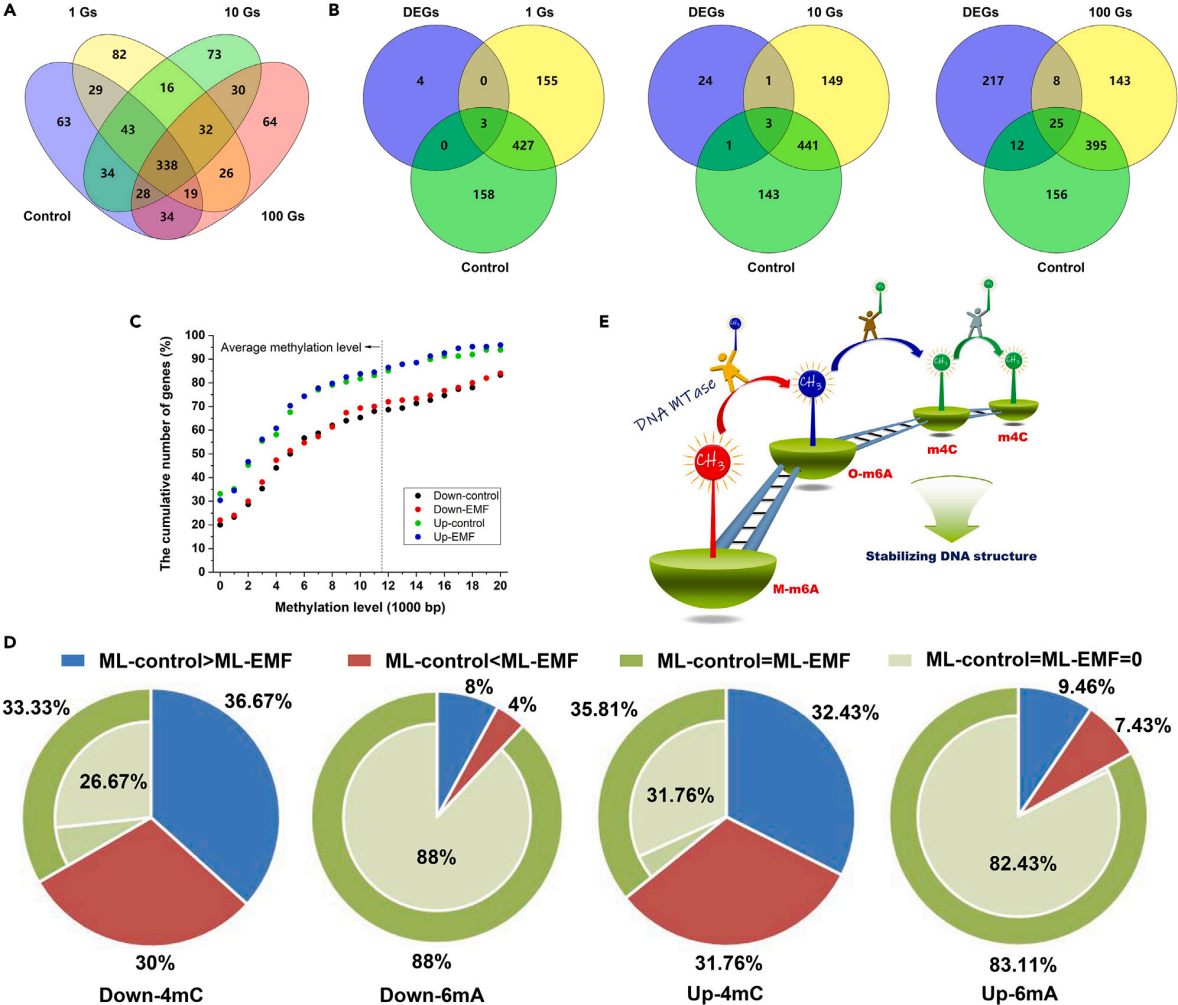
Correlation of gene expression with O-6mA and 4mC (A) Overlap analysis of O-6mA-modified genes in the four samples. (B) Overlap analysis of DEGs and O-6mA-modified genes in each comparison group. (C) 4mC methylation levels of DEGs. (D) The methylation levels of O-6mA or 4mC in upstream of the DEGs (=200 bp). ML-control, methylation level of genes in control samples; ML-EMF, methylation level of genes in ELF-EMF treated samples; Down-4mC, the methylation level of 4mC in downregulated genes; Down-6mA, the methylation level of O-6mA in downregulated genes; Up-4mC, the methylation level of 4mC in upregulated genes; Up-6mA, the methylation level of O-6mA in upregulated genes. (E) Schematic diagram for the signal pathway from M-6mA to O-6mA and then to 4mC.

The sites of M-6mA were the same in all samples, indicating that M-6mA was not related to gene expression regulation. Therefore, we mainly investigated the correlations of O-6mA and 4mC with gene expression regulation in this study. We first identified the genes containing the O-6mA modification in all samples. Overlap analysis showed that ∼300 of the O-6mA modified genes in one sample were not modified in another, and the number was greater than the number of DEGs (Figure 4A). Moreover, overlap analysis of DEGs with O-6mA-modified genes in each comparison group showed that most DEGs have no O-6mA-modification in their gene sequences (Figure 4B). These results suggest O-6mA has no direct correlation with gene expression regulation.

Next, the 4mC methylation levels of DEGs and the whole genome were compared. Result showed that among the downregulated genes, more than 68% had a lower level of 4mC modification than the methylation level of the genome, and more than 20% had no 4mC modification. Among the upregulated genes, more than 83% had a lower level of 4mC modification than the methylation level of the genome, and more than 30% had no 4mC modification (Figure 4C). These results showed that DEGs generally have a low level of 4mC modification.

Subsequently, the 4mC methylation levels of DEGs in all ELF-EMF-exposed samples were compared with the 4mC methylation levels of DEGs in the control group. Results showed that their methylation levels are correlated well with a Pearson’s correlation coefficient of 0.981 (downregulated genes, *p* = 0.001) and 0.970 (upregulated genes, *p* = 0.001) (Figure 4C), suggest that 4mC modification level had no relationship with gene expression regulation. Even so, DEGs have different 4mC distribution patterns in different samples. Considering the distribution patterns of 4mC and 6mA occur at the level of the whole genome as mentioned previously, changes in the 4mC distribution pattern of DEGs hardly serves for regulating gene expression.

In addition, the methylation levels of O-6mA or 4mC in upstream of the DEGs (=200 bp)^37^ were calculated and their effects on gene expression were explored. As for the 4mC methylation level of upregulated genes, the number of genes with higher level was nearly equal to those with lower level than control and 26% of DEGs did not show any 4mC modification. This result was the same for the 4mC methylation level of downregulated genes (Figure 4D). As for O-6mA modification, there were more than 82% DEGs without any O-6mA modification. These results showed no significant relationship between DEGs and their upstream DNA methylation level of 4mC and O-6mA, either for upregulated or downregulated genes.

In view of DNA methyltransferase is commonly analyzed to connect transcriptome analysis with DNA methylation studies, we further explored the relationship between methylation levels and gene expression levels of methyltransferase genes. There are two methyltransferase genes, GSU0227 and GSU1244, in the genome of *G. sulfurreducens*. Our results showed that, the expression level of GSU0227 was significantly downregulated in samples under 10 G and 100 G ELF-EMF treatments as compared to the control, whereas the methylation difference was almost negligible. In addition, although the difference of GSU1244 methylation level was relatively large among the four groups, the gene expression level showed no significant change (Table S4). These results further suggest that DNA methylation does not serve for gene expression regulation.

It is known that factors that play a regulatory role in cells are generally small in number, so that a small change in the number could trigger a rapid response to environmental stimuli (e.g., relatively low concentrations of Ca^2+^ can act as the second messenger of the cells, while the relatively high concentrations of Na^+^ and K^+^ cannot^38^). In this study, the number of differential methylation sites was much larger than the number of DEGs (e.g., in comparison of 1 G ELF-EMF–treated samples with control, the number of 4mC and 6mA differential modification sites was more than 4,000 times that of DEGs), suggesting DNA methylation modification should not be a regulatory factor in cell.

Organisms respond to different environments by regulating gene expression, and the regulation of gene expression requires the participation of a variety of regulatory factors. When these regulatory factors bind to target genes, the conformation of target genes will be inevitably reshaped, which is followed by the conformational change of the whole genome. Considering that DNA methylation is a genomic level modification as reasoned previously, the role of DNA methylation in organisms is more likely to stabilize the genome conformation respond to the new environment to ensure the stability of the new transcription level. That is, the DNA methylation is a response that happened after gene expression shift rather than a regulatory factor for shifting gene expression. As displayed in Figure 4E, a reshaped DNA structure was formed due to the regulation of gene expression levels associated with environmental adaptation. The reshaped DNA structure should be stabilized to ensure the continuity of the adaptive ability. One of the main strategies is DNA methylation and demethylation based on 6mA and 4mC crosstalk, that is, in the process of DNA methylation, DNA methyltransferase uses M-6mA as the reference to modify their neighboring adenine into O-6mA, then uses O-6mA as the reference to modify their neighboring cytosine into 4mC, and further uses 4mC as the reference to modify their neighboring cytosine into 4mC following the law of power function.

### Limitations of the study

Results of the present study reveal that DNA methylation is a cascade reaction at the genomic level and is not involved in gene expression regulation in *G. sulfurreducens*. Future studies to explore the DNA methylation modification mechanism and extend similar analysis to methylomes of more species should be conducted to gain a broad view of the evolutionary history and functional significance of methylation.

## STAR★Methods

### Key resources table

**Table:** 

REAGENT or RESOURCE	SOURCE	IDENTIFIER
**Bacterial and virus strains**		

*Geobacter sulfurreducens* PCA	DSMZ	DSMZ 12127

**Critical commercial assays**		

TIANamp Bacteria DNA Kit	TIANGEN	Cat#DP430
FastQuant RT Kit	TIANGEN	Cat#KR1C6-02
GoTaq® qPCR Master Mix	Promega	Cat#A6001

**Deposited data**		

DNA methylation SMRT sequencing data files and Transcriptomic sequencing data files	This paper	CNP0003188

**Other**		

ELF-EMF exposure system	Hunan Foreverelegance Co., Ltd. (China)	FE-3580AF

### Resource availability

#### Lead contact

Further information and requests for resources and information should be directed to and will be fulfilled by Zhenhua Shi (zhshi@fafu.edu.cn).

#### Materials availability

This study did not generate new unique materials.

#### Data and code availability

• DNA methylation SMRT sequencing data files and Transcriptomic sequencing data files has been deposited at CNSA. Accession number is listed in the key resources table. RT-qPCR data reported in this paper will be shared by the lead contact upon reasonable request.
• This paper does not report original code.
• Any additional information required to reanalyze the data reported in this paper is available from the lead contact upon request.

### Method details

#### Bacterial strains and growth conditions

*Geobacter sulfurreducens* PCA (=DSMZ 12127), were routinely cultured anaerobically at 30°C in mineral-based medium containing: CH_3_COONa (0.85 g/L), Fe (III) citrate (13.7 g/L), NaHCO_3_ (2.5 g/L), NH_4_Cl (0.25 g/L), NaH_2_PO_4_·2H_2_O (0.88 g/L), and KCl (0.1 g/L). The medium was adjusted to pH 6.8 and flushed with oxygen-free N_2_/CO_2_ (80/20, v/v) prior to sealing with butyl rubber stoppers and autoclaving.

#### Exposure system and sample harvest

Mid-exponential phase cultures were treated with ELF-EMF in the exposure system (FE-3580AF, Hunan Foreverelegance Co., Ltd., China) for 2 h.^30^ The control samples were put into a system comprised the same components as the exposure system, just turning off the switch that generates the ELF-EMF. All samples were harvested at the mid-exponential phase, and their pellets were frozen in liquid nitrogen and stored at −80°C before DNA and RNA extraction.

#### Single-molecule real-time sequencing

SMRT sequencing and data analysis were carried out with the help of Berry Genomics Co.,Ltd. (Beijing, China). In brief, DNA of three biological replicates was isolated with the TIANamp Bacteria DNA Kit (TIANGEN, Cat. No. DP302-02), and then take 2 μg DNA from each batch and mix them into one sample. Libraries of replicates of *G. sulfurreducens* were prepared for SMRT sequencing using a previously described library construction.^39^^,^^40^ These libraries were sequenced to a mean genome coverage depth of >1000× on the PacBio Sequel II platform (Pacific Biosciences, Menlo Park, CA, USA) using standard protocols^40^ (Figure S2A) and average read lengths were >20 kbp (Figure S2B). Read quality score lower than 0.8 was removed in all libraries. Sequencing reads were processed and mapped to the reference sequences (RefSeq AE017180.2) using the software program BLASR.^41^ BLASR generates a Mapping QV representing the confidence that the read maps are unique to the selected genomic interval. Base modification and motif detection was performed using the modification and motif detection protocol in the software program SMRTPipe v.1.3.3. Positions with coverage of >25× and kinetic scores of QV ≥ 100 were considered modified. The QV is the −10 ∗ log (*p* value), where the *p* value was determined from a *t* test between the sample and the in silico model (Table S5).

#### RNA sequencing and data analysis

RNA sequencing and data analysis were carried out with the help of Personal Biotechnology Co.,Ltd (Shanghai, China). In brief, approximately 3 μg of total RNA was used to construct sequencing libraries with an NEBNext Ultra Directional RNA Library Prep Kit for Illumina (NEB, Ipswich, MA, USA), per the manufacturer’s instructions. The libraries, with three biological replicates per sample, were then sequenced on an Illumina HiSeq-1500 high-throughput sequencing platform. Raw reads were first processed through in-house perl scripts to remove reads containing adapter or ploy-N or with base quality score lower than 20. All remaining clean reads were mapped to the reference genome (Table S5) using Bowtie2 (http://bowtie-bio.sourceforge.net/index.shtml), and read numbers mapped to each gene were calculated using the HTSeq 0.6.1p2 (Table S5). Differential expression analyses were performed using the DESeq of R package. Transcripts with an adjusted *p*-value < 0.05 were considered to be DEGs. The topGO was used for GO enrichment analysis, and KOBAS software was used to test the statistical enrichment of the DEGs in KEGG pathways.^42^^,^^43^ GO terms or KEGG pathways with a corrected *p*-value < 0.05 were considered significantly enriched.

#### RNA extraction and qRT-PCR measurements

Total RNA of six biological replicates was isolated with the TIANamp Bacteria DNA Kit (TIANGEN, Cat. No. DP430), and then reversely transcribed to cDNA in accordance with the manufacturer’s instructions of FastQuant RT Kit (with gDNase) (TIANGEN, Cat. No. KR1C6-02). RNA concentrations were measured using an ND-1000 spectrophotometer (Nanodrop Technology, Wilmington, DE, USA).

Gene-specific primers were designed using Primer 5.0 software, and two genes (*rec*A and *gap*A) were selected as internal references (Table S6).^20^ QRT-PCR was performed using the GoTaq qPCR Master Mix (Promega, Cat. No. A6001) and the Roche LightCycle 96 sequence detection system (Roche, Switzerland). Relative fold change in gene expression for each gene was calculated using normalized CT values. Relative expression was standardized to *rec*A and *gap*A expression, yielding similar fold differences.

### Quantification and statistical analysis

Differences in 4mC and 6mA modification levels and differences in mRNA levels were statistically analyzed by one-way ANOVA. The results of each group were compared using Dunnett’s two-sided test (SPSS 24 Software, SPSS Inc., Chicago, IL, USA). Significance levels were set to *p* < 0.05 (∗ represents *p* < 0.05, ∗∗ represents *p* < 0.01, # represents *p* < 0.001). Data are expressed as means ± standard errors of the means (SEM).

In order to explore the correlation of 4mC and 6mA, the distances of these neighboring modifications and the frequency of each distance were completed using Excel 2010.^33^ The allometric fit of frequency and distance, the fitting formulae, and the values of R-square were obtained using OriginPro 8.0. Correlation analyses were performed using SPSS software version 24.0 (SPSS Inc., Chicago, IL, USA).
