# Supplementary material for: Crosstalk between 6-methyladenine and 4-methylcytosine in Geobacter sulfurreducens exposed to extremely low-frequency electromagnetic field
Source: iScience. 2024 Jul 27;27(9):110607. doi: 10.1016/j.isci.2024.110607 (PMC11388800; doi:10.1016/j.isci.2024.110607)
Supplement: Document S1. Figures S1, S2, and Tables S1–S6 [file mmc1.pdf]

iScience, Volume *27*

Supplemental information

Crosstalk between 6-methyladenine and 4-methylcytosine in *Geobacter sulfurreducens* exposed to extremely low-frequency electromagnetic field

Zhenhua Shi, Yingrong Zhang, Wanqiu Chen, and Zhen Yu

Supplemental Figure S1 Volcano plot of DEGs. The number of DEGs in the comparison of the exposure groups also increased with the increase in ELF-EMF intensity. Related to Figure 3.

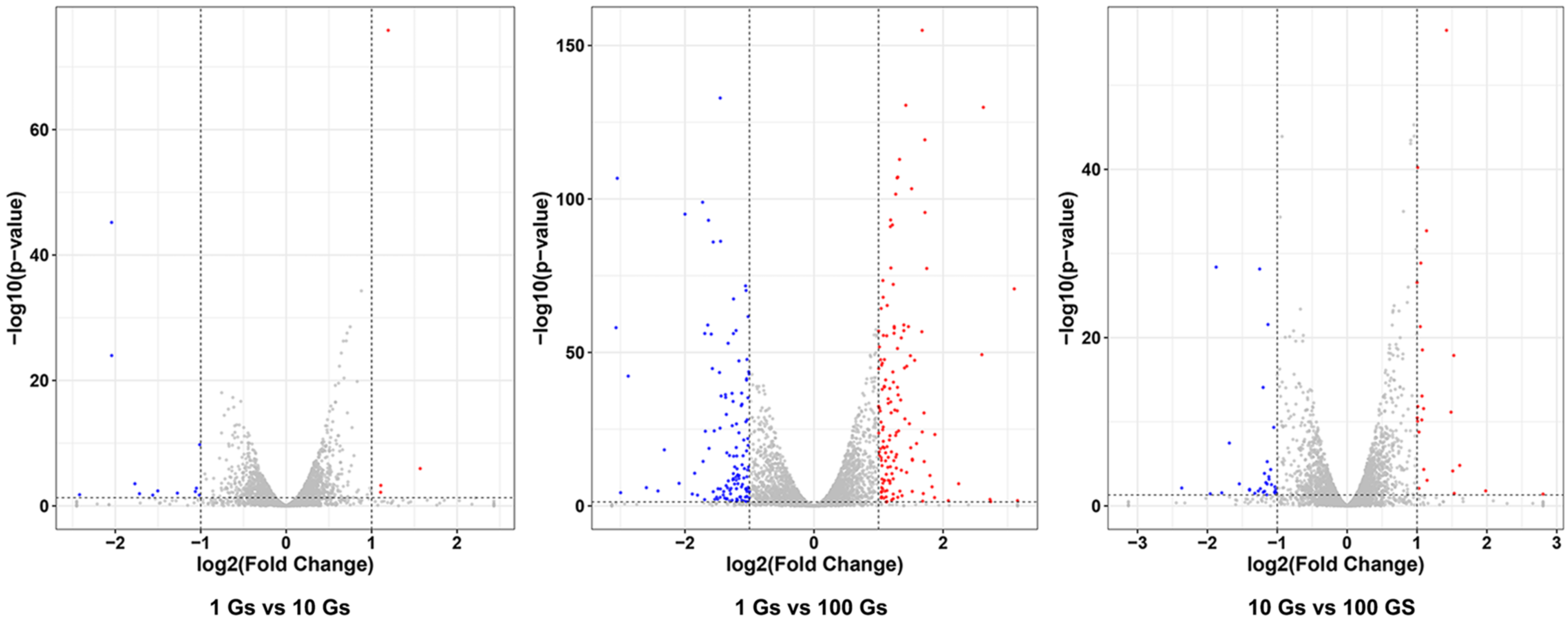

Supplemental Figure S2 Description of SMRT sequencing data sets used. a These libraries were sequenced to a mean genome coverage depth of > 1000× on the PacBio Sequel II platform. **b** Average read lengths were > 20 kbp in all libraries. Related to STAR Methods.

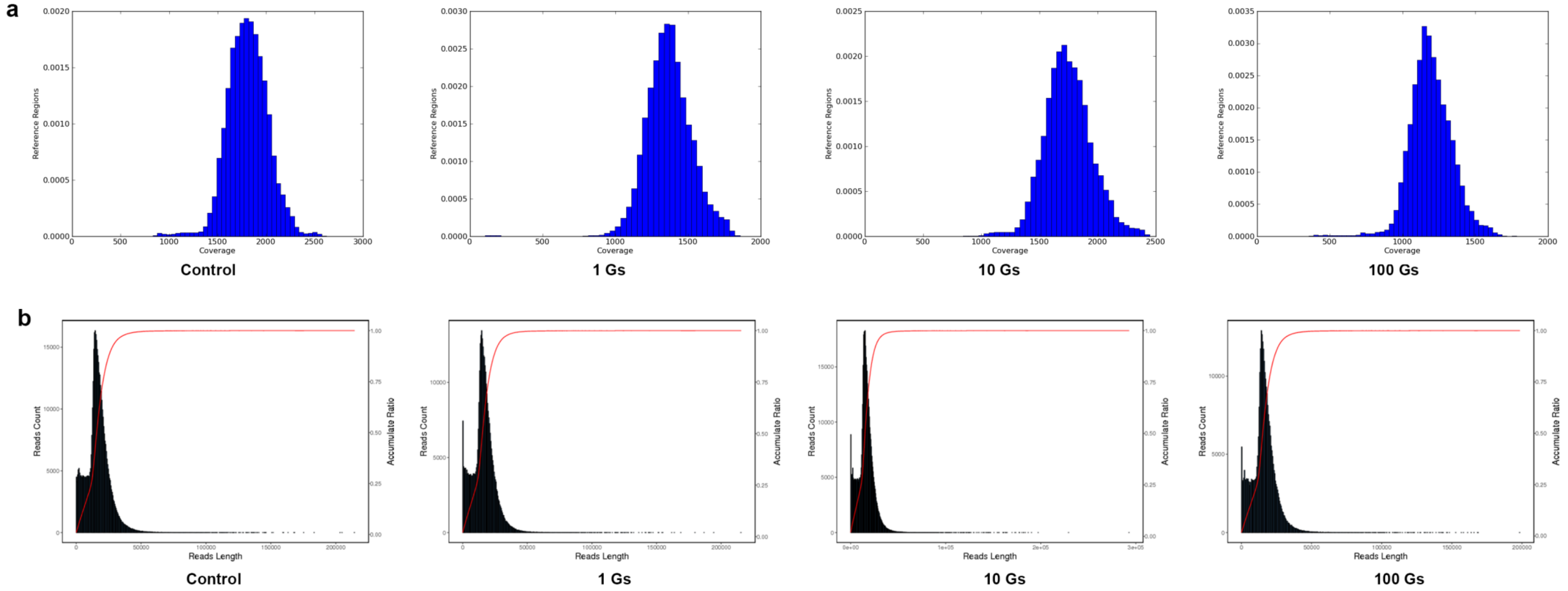

Supplemental Table S1 Expression changes of six genes determined by RNA-seq and qRT-PCR. qRT-PCR1 used rec A (GSU0856) as internal references, while qRT-PCR2 used gap A (GSU1629) as internal references. *P < 0.05, **P < 0.01, and #P < 0.001. Related to Figure 3.

Gene	Comparison group	Fold change			Correlation (Sig.)		
		qRT-PCR1	qRT-PCR2	Transcriptome	qRT-PCR1 vs qRT-PCR2	qRT-PCR1 vs Transcriptome	qRT-PCR2 vs Transcriptome
GSU1069	CK vs 100 Gs	0.513056*	1.762778*	2.333884			
GSU1496	1 Gs vs 100 Gs	0.013333#	2.148056#	2.2095771			
GSU1760	1 Gs vs 100 Gs	1.515833**	3.650556**	2.8262378			
GSU1760	10 Gs vs 100 Gs	0.828056**	2.081389*	2.0966343			
GSU1761	1 Gs vs 100 Gs	-3.25583**	-1.12111#	-0.461157	0.962 #	0.888**	0.834**
GSU1873	1 Gs vs 100 Gs	2.254167**	4.388889*	2.157815			
GSU3291	CK vs 100 Gs	2.334722#	3.584444#	2.4605337			
GSU3291	1 Gs vs 100 Gs	1.665833#	3.800556#	2.7068172			
GSU3291	10 Gs vs 100 Gs	1.216389#	2.469722#	2.8803449			

Supplemental Table S2 GO analysis of DEGs. BP, biological process; CC, cellular component; MF, molecular function. Related to Figure 3.

Groups	Category	GO.ID	Term	Number of up-regulated genes	Number of down-regulated genes	*P*-value
Control vs 1Gs	BP	GO:0015709	thiosulfate transport	0	1	0.0019
	BP	GO:0008272	sulfate transport	0	1	0.0097
	BP	GO:1902358	sulfate transmembrane transport	0	1	0.0097
	BP	GO:0072348	sulfur compound transport	0	1	0.0116
	BP	GO:0098661	inorganic anion transmembrane transport	0	1	0.0232
	BP	GO:0015698	inorganic anion transport	0	1	0.0403
	CC	GO:0030288	outer membrane-bounded periplasmic space	0	1	0.031
	CC	GO:0042597	periplasmic space	0	1	0.048
	MF	GO:0008271	secondary active sulfate transmembrane transporter activity	0	1	0.0027
	MF	GO:0004779	sulfate adenylyltransferase activity	0	1	0.0041
	MF	GO:0004781	sulfate adenylyltransferase (ATP) activity	0	1	0.0041
	MF	GO:0015419	ATPase-coupled sulfate transmembrane transporter activity	0	1	0.0054
	MF	GO:0015116	sulfate transmembrane transporter activity	0	1	0.0067
	MF	GO:1901682	sulfur compound transmembrane transporter activity	0	1	0.0067
	MF	GO:0070566	adenylyltransferase activity	0	1	0.0175
	MF	GO:0043225	ATPase-coupled anion transmembrane transporter activity	0	1	0.0202
	MF	GO:0015103	inorganic anion transmembrane transporter activity	0	1	0.0255
	MF	GO:0015291	secondary active transmembrane transporter activity	0	1	0.0374

**Groups**	**Category**	**GO.ID**	**Term**	**Number of up-regulated genes**	**Number of down-regulated genes**	***P*-value**
Control vs 10Gs	BP	GO:0000097	sulfur amino acid biosynthetic process	0	3	0.0003
	BP	GO:0000096	sulfur amino acid metabolic process	0	3	0.00049
	BP	GO:0019344	cysteine biosynthetic process	0	2	0.00095
	BP	GO:0006534	cysteine metabolic process	0	2	0.00175
	BP	GO:0006790	sulfur compound metabolic process	0	4	0.00178
	BP	GO:0009086	methionine biosynthetic process	0	2	0.00405
	BP	GO:0009070	serine family amino acid biosynthetic process	0	2	0.00476
	BP	GO:0044272	sulfur compound biosynthetic process	0	3	0.00573
	BP	GO:0006555	methionine metabolic process	0	2	0.00635
	BP	GO:0006183	GTP biosynthetic process	1	0	0.00827
	CC	GO:0042597	periplasmic space	0	2	0.018
	CC	GO:0009326	formate dehydrogenase complex	1	0	0.025
	MF	GO:0016835	carbon-oxygen lyase activity	0	3	0.0041
	MF	GO:0004550	nucleoside diphosphate kinase activity	1	0	0.0077
	MF	GO:0004604	phosphoadenylyl-sulfate reductase (thioredoxin) activity	0	1	0.0077
	MF	GO:0016622	oxidoreductase activity, acting on the aldehyde or oxo group of donors, cytochrome as acceptor	1	0	0.0077
	MF	GO:0043546	molybdopterin cofactor binding	1	0	0.0077
	MF	GO:0047111	formate dehydrogenase (cytochrome-c-553) activity	1	0	0.0077
	MF	GO:0003688	DNA replication origin binding	1	0	0.0153
	MF	GO:0003961	O-acetylhomoserine aminocarboxypropyltransferase activity	0	1	0.0153
	MF	GO:0003962	cystathionine gamma-synthase activity	0	1	0.0153
	MF	GO:0004121	cystathionine beta-lyase activity	0	1	0.0153

Groups	Category	GO.ID	Term	Number of up-regulated genes	Number of down-regulated genes	*P*-value
Control vs 100Gs	BP	GO:0015628	protein secretion by the type II secretion system	0	5	0.0022
	BP	GO:0098776	protein transport across the cell outer membrane	0	5	0.0022
	BP	GO:0006812	cation transport	8	4	0.0024
	BP	GO:0006221	pyrimidine nucleotide biosynthetic process	3	2	0.0053
	BP	GO:0030001	metal ion transport	6	2	0.0057
	BP	GO:0009263	deoxyribonucleotide biosynthetic process	1	2	0.0085
	BP	GO:0006811	ion transport	10	6	0.0104
	BP	GO:0006220	pyrimidine nucleotide metabolic process	3	2	0.0108
	BP	GO:0009162	deoxyribonucleoside monophosphate metabolic process	1	1	0.0128
	BP	GO:0009262	deoxyribonucleotide metabolic process	1	2	0.0184
	CC	GO:0030288	outer membrane-bounded periplasmic space	1	4	0.004
	CC	GO:0042597	periplasmic space	1	4	0.029
	CC	GO:0015627	type II protein secretion system complex	0	3	0.03
	MF	GO:0016861	intramolecular oxidoreductase activity, interconverting aldoses and ketoses	0	4	0.003
	MF	GO:0016860	intramolecular oxidoreductase activity	0	4	0.0044
	MF	GO:0008882	[glutamate-ammonia-ligase] adenylyltransferase activity	0	2	0.0045
	MF	GO:0046873	metal ion transmembrane transporter activity	5	2	0.0115
	MF	GO:0016435	rRNA (guanine) methyltransferase activity	0	2	0.0128
	MF	GO:0015036	disulfide oxidoreductase activity	0	4	0.0149
	MF	GO:0019205	nucleobase-containing compound kinase activity	2	1	0.0185
	MF	GO:0016798	hydrolase activity, acting on glycosyl bonds	1	3	0.0188
	MF	GO:0016597	amino acid binding	2	2	0.0234
	MF	GO:0004355	glutamate synthase (NADPH) activity	0	2	0.0246

Supplemental Table S3 KEGG analysis of DEGs. Related to Figure 3.

**Groups**	**PathwayID**	**Pathway**	**Number of up-regulated genes**	**Number of down-regulated genes**	***P*-value**
Control vs 1Gs	ko00261	Monobactam biosynthesis	0	1	0.0113707
	ko00450	Selenocompound metabolism	0	1	0.0170236
	ko00920	Sulfur metabolism	0	1	0.0189031
Control vs 10Gs	ko00450	Selenocompound metabolism	0	2	0.002923
	ko00920	Sulfur metabolism	0	2	0.0036337
	ko00270	Cysteine and methionine metabolism	0	2	0.0206362
	ko00680	Methane metabolism	1	1	0.0240348
	ko00230	Purine metabolism	1	1	0.0334588
Control vs 100Gs	ko00230	Purine metabolism	5	2	0.0055037
	ko00670	One carbon pool by folate	3	0	0.0404159

Supplemental Table S4 DNA methylation level and gene expression level of methyltransferase genes. "↓" means down regulation. Related to Figure 4.

Genes	Methylation region	Sequence length (bp)	The number of 4mC sites				The number of 6mA sites				Gene expression comparison (*P*-value)		
			Control	1 Gs	10 Gs	100 Gs	Control	1 Gs	10 Gs	100 Gs	Control vs 1 Gs	Control vs 10 Gs	Control vs 100 Gs
GSU0227	Gene sequence	918	0	1	1	1	0	0	0	1	0.65	0.01 ↓	0.00 ↓
	Upstream of gene sequence	200	0	1	0	0	0	0	0	0			
GSU1244	Gene sequence	564	2	13	13	0	1	0	0	0	0.69-	00.75	0.82
	Upstream of gene sequence	200	0	1	0	0	2	0	0	0			

Supplemental Table S5 Links used in the manuscript. Related to STAR Methods.

website	link
Determination of QV and P value	https://www.pacb.com/applications/base_modification/index.html
Reference genome	https://www.ncbi.nlm.nih.gov/nuccore/NC_002939.5?report=genbank&to=3814128
HTSeq 0.6.1p2	https://htseq.readthedocs.io/en/master

Supplemental Table S6 Primers used in qRT-PCR examination. Related to STAR Methods.

No.	Genes	Forward primer（5'- 3'）	Reverse primer （5'- 3'）
1	GSU0856	GCTGAGCGGGTTGACGATG	GCGAGTATTTGGCGGACGA
2	GSU1629	ACGGGAGAAGTTCGGGATT	GGAGAAGGGCATTGAGATTG
3	GSU1069	ACTGGGAAAGGGCGAACA	AGCTCCACCGGAAGAAGA
4	GSU1496	CGGCGTATCGTGTCAAGG	CTTTCGGGCGGATAGGTT
5	GSU1760	CACCCACAAACGACACTCT	CGTAATCCTTGCCGAAAT
6	GSU1761	CCAGACCAGGACTGTATCGG	ACGTGAGGGTTGAAAGGAAC
7	GSU1873	CCCTTGATGCGGCTGAG	GGCGGCAAAGAGGAGGT
8	GSU3291	GAACTGGCGGCGAACCTCT	CCTCCTACGTGGACGAACTGAA
